# From prevention to management: Exploring the impact of diet on multiple sclerosis

**DOI:** 10.1515/tnsci-2025-0371

**Published:** 2025-05-14

**Authors:** Dalya Koukach, Maryam Aljumaily, Noora Al-Attiyah, Rawdhah Al-Amer, Yasmine Attia, Reema Tayyem

**Affiliations:** Department of Nutrition Sciences, College of Health Science, Qatar University, Doha, 2713, Qatar

**Keywords:** multiple sclerosis, dietary patterns, nutrients, microbiome

## Abstract

Multiple sclerosis (MS) is a chronic immune-mediated disease of the central nervous system characterized by neuroinflammation and progressive neurodegeneration. Growing evidence suggests that dietary interventions may influence MS progression and symptom management by modulating inflammation, oxidative stress, and gut microbiota composition. This narrative review examines the effects of the Mediterranean, plant-based, ketogenic, Wahls, Swank, intermittent fasting, and gluten-free diets, alongside key nutrients such as omega-3 fatty acids, vitamin D, polyphenols, and antioxidants. Among these, Mediterranean and plant-based diets have shown the most consistent benefits, including reductions in fatigue, improved quality of life, and modulation of inflammatory markers. The Wahls and Swank diets show promise but are primarily supported by studies from their respective research groups, raising concerns about long-term adherence and nutritional adequacy. The ketogenic diet and intermittent fasting have yielded mixed findings, with some studies suggesting benefits for fatigue and neuroprotection, while others highlight potential metabolic risks. The gluten-free diet and omega-3 supplementation lack robust evidence, with inconsistent findings across studies. Additionally, ultra-processed foods and diets high in saturated fats have been associated with increased inflammation and greater MS severity. Despite promising findings, limitations such as small sample sizes, short follow-up durations, and study design inconsistencies prevent definitive conclusions. Future research should prioritize large-scale, long-term randomized controlled trials to establish the efficacy, safety, and sustainability of dietary interventions in MS management. Mechanistic studies and standardized dietary protocols are also needed to better understand the role of diet in MS progression and symptom control.

## 1 Introduction

Multiple sclerosis (MS) is a chronic immune-mediated disease of the central nervous system (CNS), characterized by inflammation, gliosis, and demyelination that progressively lead to neurological deterioration. Common symptoms include visual impairments, fatigue, cognitive decline, numbness, and disruptions in sensory and motor functions [1]. Globally, MS affects over 2.8 million individuals, with prevalence rates exceeding 100 cases per 100,000 in North America, Europe, and Australia, while rates in equatorial regions are typically below 30 per 100,000 [2]. In Qatar, the prevalence is estimated at 64.6 per 100,000 individuals [3]. MS manifests in two primary forms – relapsing and progressive – further categorized into relapsing-remitting MS (RRMS), secondary progressive MS (SPMS), and primary progressive MS (PPMS) [4]. RRMS, the most common subtype, affects approximately 85% of individuals and is characterized by episodes of neurological dysfunction followed by varying degrees of remission. As relapse frequency declines over time, symptoms may gradually worsen, leading to SPMS in approximately 15–30% of individuals [5]. In contrast, PPMS, which affects 10–15% of individuals, is characterized by a steady progression of symptoms from onset, independent of relapse occurrence [6].

Despite differences in disease progression, all MS subtypes share similar underlying mechanisms, with both inflammation and neurodegeneration occurring throughout the disease continuum [6]. Epidemiologically, MS demonstrates notable sex-based differences, with women nearly three times more likely than men to develop RRMS, typically around the age of 30. By contrast, PPMS affects both sexes equally and has a mean onset age of 40 years [6]. Diagnosing MS follows the 2017 McDonald criteria, which for RRMS requires evidence of clinical attacks and tissue damage occurring at different times and locations. PPMS diagnosis demands confirmation of progressive disability over at least 12 months alongside spatial lesion dissemination, while SPMS is diagnosed when disability progression follows an initial RRMS diagnosis [4]. These diagnostic criteria, along with MS’s complex etiology, highlight its multifactorial nature, involving chronic CNS inflammation driven by dysregulated T-cell activation. This immune response damages myelin and oligodendrocytes, with additional contributions from macrophages and lymphocytic infiltration [7].

Although genetic predisposition plays a significant role in MS development, environmental factors – including infections, smoking, obesity, vitamin D deficiency, and diet – are equally critical in disease onset and progression. Conversely, lifestyle modifications such as regular physical activity, a balanced diet, and weight management have demonstrated anti-inflammatory effects that may slow disease progression [8]. Standard treatment includes disease-modifying therapies such as glatiramer acetate, natalizumab, and mitoxantrone, which help reduce MRI lesion activity in the short term and slow disease progression [1]. However, pharmacological interventions alone often prove insufficient, highlighting the potential role of dietary strategies in comprehensive MS management. Anti-inflammatory diets, such as the Mediterranean diet, and restrictive regimens like the ketogenic and gluten-free diets, have shown potential benefits. Specific nutrients, including vitamin D, omega-3 fatty acids, antioxidants, and polyphenols, may also help reduce inflammation and slow disease progression, leading to improved outcomes [9]. In contrast, diets high in ultra-processed foods (UPFs), saturated fats (SFAs), and *trans* fats – common in Western diets – may exacerbate inflammation, gut dysbiosis, and neurodegeneration [10]. A deeper understanding of dietary patterns in MS could improve prevention and intervention strategies. Therefore, this narrative review explores how dietary interventions can influence MS prevention, progression, and symptom management, with a focus on key dietary patterns and nutrients.

## 2 Methods

A literature search using PubMed, ScienceDirect, Scopus, and Google Scholar was conducted to identify studies on dietary interventions for the prevention and management of MS. Search terms included antioxidants, biotin, curcumin, diet, dietary patterns, dietary supplements, egg, gluten-free diet, gut-brain axis, inflammation, immune system modulation, intermittent fasting (IF), ketogenic diet, low-fat diet, Mediterranean diet, microbiome, MS, neuroprotection, omega-3 fatty acids, oxidative stress, plant-based diet, polyphenols, pro- and prebiotics, resveratrol (RSV), Swank diet, vitamin C, vitamin D, vitamin E, and Wahls diet.

Studies published before 2012 were excluded to ensure relevance to current practices, as significant changes in MS research, diagnostic criteria, and dietary intervention strategies occurred around this time. Cohort, cross-sectional, and case–control studies, as well as systematic reviews and meta-analyses, were considered. Each author conducted the literature search independently, selecting studies aligned with the review’s objectives.

Table 1 summarizes the search strategy.

**Table 1:** The search strategy summary

Items	Specification
Date of search	November–December; 2024
Databases and other sources searched	PubMed; ScienceDirect; Scopus; and Google Scholar
Search terms used	Antioxidants; biotin; curcumin; diet; dietary patterns; dietary supplements; egg; gluten-free diet; gut-brain axis; inflammation; immune system modulation; IF; ketogenic diet; low-fat diet; Mediterranean diet; microbiome; MS; multiple sclerosis; neuroprotection; omega-3 fatty acids; oxidative stress; plant-based diet; polyphenols; pro- and prebiotics; RSV; Swank diet; vitamin C; vitamin D; vitamin E; Wahls diet
Timeframe	2012–2024
Inclusion and exclusion criteria	Studies from various designs; from clinical trials; cohort, cross-sectional, and case-control observational studies, systematic reviews, and meta-analyses
	The articles were published in the English language
Selection process	Each author conducted the literature search independently

## 3 Diet and MS

**Figure 1:**
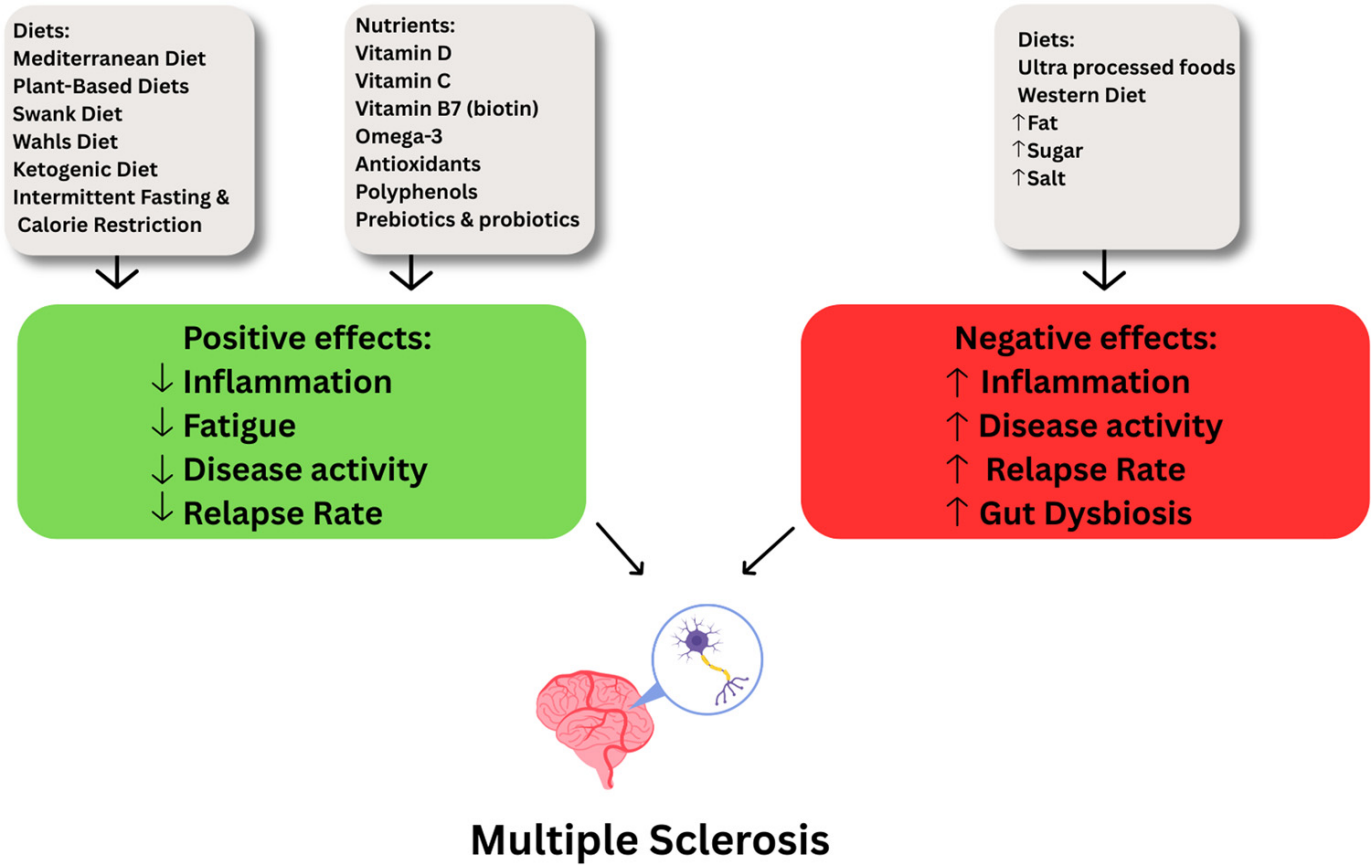
Dietary and nutritional influences on multiple sclerosis progression.

Diet plays a key role in MS, affecting immune function, inflammation, and neurodegeneration (Figure 1) [5]. While no single diet has been established as a definitive treatment for MS, emerging evidence suggests that certain dietary patterns may help manage symptoms and slow disease progression [8,9]. Anti-inflammatory and neuroprotective diets, such as Mediterranean and plant-based diets, have been linked to reduced fatigue, improved quality of life (QoL), and modulation of inflammatory markers [10]. Meanwhile, dietary patterns that involve specific restrictions, such as the Swank and Wahls diets, as well as metabolic-focused strategies like the ketogenic diet and IF, have been explored for their effects on immune regulation, oxidative stress, and symptom management [9].

In contrast, diets high in SFAs, *trans* fats, UPFs, and refined sugars may exacerbate inflammation, gut dysbiosis, and neurodegeneration, potentially worsening MS symptoms [11]. Beyond dietary components, the autonomic nervous system also influences immune function. Certain dietary factors, particularly those high in pro-inflammatory compounds, may indirectly affect immune regulation through the cholinergic anti-inflammatory pathway, which modulates systemic inflammation via vagal nerve stimulation [12]. High intake of SFAs and UPFs can disrupt gut microbiota, reducing beneficial species like *Bifidobacteria* and *Lactobacilli* while promoting inflammation. This imbalance impairs short-chain fatty acid (SCFA) production, increases intestinal permeability, and facilitates endotoxin translocation, triggering systemic inflammation [13].

*Trans* fats, commonly found in hydrogenated oils, processed snacks, and fast foods, have also been linked to inflammation, while red meat, which is rich in arachidonic acid, may promote inflammation through T helper 17 cells (Th17 cell) activation [14]. Excessive sugar intake raises insulin levels, which may indirectly enhance arachidonic acid metabolism and contribute to systemic inflammation. Additionally, high salt intake and cow’s milk proteins have been implicated in immune dysregulation and MS pathogenesis [14].

In addition to inflammation, oxidative stress plays a key role in MS progression by generating reactive oxygen species (ROS) that damage neurons, degrade myelin, and amplify inflammatory responses. Antioxidants such as vitamins C and E, omega-3 fatty acids, and polyphenols may help counteract these effects. Among them, curcumin has demonstrated neuroprotective properties by reducing CNS inflammation and oxidative damage [15,16]. Although these dietary interventions show promise, further research is needed to clarify their long-term impact on MS progression and clinical outcomes [9].

## 4 Dietary patterns and MS

### 4.1 Mediterranean diet

The Mediterranean diet has been widely studied for its potential role in managing and preventing MS, with multiple studies suggesting benefits in symptom reduction and disease modulation.

A prospective observational study analyzed dietary patterns among 163 MS patients, categorizing them into three groups: Western, plant-rich, and varied [17]. The plant-rich diet, which closely resembles the Mediterranean diet, demonstrated significant symptom reductions (19–90%, *p* = 0.012), including pain (90.1%, *p* = 0.022) and bladder dysfunction (90%, *p* = 0.031). However, the small sample size (*n* = 17) and reliance on self-reported data limits the generalizability of these findings [17]. Similarly, a randomized controlled trial (RCT) investigated the effects of a Mediterranean-like diet in 72 patients with RRMS over a 1-year period [18]. The intervention group adhered to a Mediterranean-like diet, which emphasized a high intake of fruits, vegetables, whole grains, olive oil, and fish, while the control group followed a standard diet [18]. The study found that the Mediterranean-like diet significantly improved fatigue scores (final fatigue score: 33.93 vs 37.98, *p* < 0.001) and reduced body mass index (BMI) (27.29–26.0, *p* = 0.02), suggesting potential benefits for physical health and QoL in RRMS patients [18]. However, cognitive improvements were limited. Interestingly, the standard diet group outperformed the Mediterranean-like diet group on the Brief Visuospatial Memory Test-Revised (mean score: 23.73 vs 20.56, *p* = 0.020), raising questions about the diet’s impact on cognitive function. This study has several limitations. First, cultural adaptations to the Mediterranean-like diet may restrict the generalizability of findings to other populations. Second, the absence of depression assessments limited a comprehensive evaluation of mental health outcomes, which are crucial in MS. Finally, the study did not investigate potential mechanisms, such as inflammation, oxidative stress, or gut microbiota changes, that may underlie the observed effects. A pilot study involving 15 early-stage MS patients over 12 weeks found significant improvements in QoL (80.3–87.5, *p* = 0.02), cognitive performance (56.4–63.1, *p* = 0.006), and fatigue (32.5–21.9, *p* = 0.005) [19]. Despite promising results, the study’s small sample size and lack of a control group limit its ability to establish causality and generalizability to a wider MS population [19]. A 12-month RCT carried out by Bagheri et al. on 100 MS patients found significant improvements in fatigue levels (30% reduction, *p* < 0.001) and cognitive scores (15% improvement, *p* < 0.01) following adherence to a Mediterranean-like diet [20]. QoL also improved by 20% (*p* < 0.005). However, the study lacked imaging data to assess neurological changes. A systematic review and network meta-analysis of randomized trials assessed the efficacy of eight dietary interventions: low-fat, Mediterranean, ketogenic, anti-inflammatory, Paleolithic, fasting, calorie restriction, and a control diet on fatigue and QoL in MS [21]. The results indicated that the Mediterranean and Paleolithic diets led to a greater reduction in fatigue compared to the control diet. These diets also showed greater improvements in both physical and mental QoL [21]. However, limitations include moderate to high risk of bias, a small number of studies, and limited generalizability due to sample size constraints. While these findings provide valuable insights, they require validation through higher-quality RCTs before being integrated into clinical practice [21].

A case–control study by Mirza et al. examined the relationship between Mediterranean diet adherence and pediatric-onset MS [22]. The study assessed adherence using the alternate Mediterranean diet (aMED) score, a dietary index that awards points based on intake of key food groups (e.g., fruits, vegetables, whole grains, legumes, nuts, and fish) while awarding fewer points for higher consumption of red and processed meats [22]. Findings indicated that each one-point increase in aMED score was associated with 34% lower odds of MS (odds ratio [OR] = 0.66, *p* = 0.011). The study also highlighted the role of fiber intake, showing that each additional gram of fiber consumed per day was linked to a 13% lower likelihood of MS (*p* = 0.011) [22]. The potential protective effects of the Mediterranean diet may, in part, be mediated by its influence on gut microbiota. It was observed in the study that individuals with higher adherence to the Mediterranean diet exhibited distinct microbial compositions, suggesting that dietary-induced shifts in gut bacteria may influence inflammatory pathways and immune modulation in MS [22]. While the exact mechanisms remain unclear, this aligns with broader evidence linking diet, gut microbiota, and neuroinflammation. However, as a case–control study, its retrospective design and small sample size limit its ability to establish causality. Further research is needed to clarify specific microbial shifts and their implications for immune modulation in MS.

Across these studies, the Mediterranean diet consistently demonstrates potential in reducing fatigue, improving QoL, and modulating inflammation. However, findings are constrained by limitations such as small sample sizes, short intervention periods, and reliance on self-reported data. Future longitudinal trials with larger, more diverse populations are needed to validate these effects and determine the long-term impact of the Mediterranean diet on MS progression and prevention.

### 4.2 Plant-based diets

Plant-based diets are being explored for their potential role in MS management, particularly due to their anti-inflammatory properties and ability to modulate gut microbiota. Studies indicate that individuals with MS exhibit gut dysbiosis [23]. A case-control study of 148 MS patients and 148 healthy controls found significant differences in gut microbiota composition (*p* < 0.001). Among 61 bacterial species with altered abundance, 31 were more prevalent in MS patients, with some directly linked to inflammation markers (*p* < 0.05) [24]. These findings suggest that gut microbiota imbalances may contribute to MS-related inflammation, reinforcing the potential benefits of plant-based diets – rich in fiber, vitamins, and phytochemicals – in improving gut health and reducing inflammation [24]. A 12-month RCT investigated a low-fat, plant-based diet in 61 RRMS patients [25]. Although MRI and relapse rates remained unchanged, diet participants showed notable monthly improvements in fatigue severity scale (FSS, 0.06 points), mental fatigue impact scale (MFIS, 0.23 points), and BMI (0.18 kg/m²) [25]. Mental QoL also trended positively, with a 0.298-point monthly improvement on the Short Form-36 mental scale (0.298 points/month; *p*-adjusted = 0.077) [25]. Although the observed effect trended positively, the minimal clinically important difference (MCID) for the SF-36 mental component has not been well established for MS. However, in other chronic conditions, MCID values range from 4 to 10 points, including multiple chronic diseases [26] and orthopedic oncology patients [27]. Since the observed improvement in this study was 0.298 points per month, it is unlikely to be clinically meaningful.

Another study investigated dietary habits and health outcomes in 2,469 MS patients through an online survey [28]. Healthier diets – high in fruits, vegetables, and whole grains and low in sugar, dairy, and processed foods – were linked to significant QoL improvements [28]. A 10-point increase in diet quality scores correlated with a nearly 6.0-point rise in physical QoL and a 5.0-point rise in mental QoL. In RRMS patients, healthier diets were associated with a lower relapse rate and 30% reduced odds of higher disability levels [28]. In a more controlled study, an RCT examined lifestyle behaviors in 857 MS patients enrolled in an online course [29]. Participants who adopted at least four healthy behaviors – including a plant-based diet, regular physical activity, and omega-3 and vitamin D supplementation – reported a 9.0-point higher mental QoL and a 9.5-point higher physical QoL [29]. Additionally, compared to those engaging in fewer lifestyle interventions, these individuals had a 23% lower prevalence of fatigue and a 56% lower prevalence of moderate disability [29].

While these studies suggest potential benefits of plant-based diets in reducing inflammation and improving QoL in MS, limitations such as self-reported dietary data, short trial durations, and small sample sizes reduce the strength of these findings. Future large-scale, well-controlled trials are needed to confirm these effects and assess their long-term impact on MS progression.

### 4.3 Swank diet

The Swank diet, originally developed by Dr. Roy Swank, is a low-fat dietary approach that limits SFA intake to ≤15 g/day while allowing up to 50 g of unsaturated fat per day. It promotes whole grains, fruits, vegetables, and lean protein while restricting processed foods, dairy, and red meat. The diet’s proposed benefits stem from its ability to reduce SFA intake, which may influence inflammation, lipid metabolism, and myelin integrity. However, long-term clinical outcomes remain uncertain, and concerns about potential micronutrient deficiencies persist.

A 7.5-year longitudinal study analyzed self-reported adherence to the Swank diet among 671 MS patients [30]. Participants who consistently adhered to the diet reported a 20–30% reduction in fatigue and depression severity [30]. However, the study’s reliance on self-reported adherence introduces potential bias, and only 7% of participants strictly followed the diet, limiting the generalizability of findings. Additionally, the study had a high attrition rate (73%), reducing its ability to provide definitive conclusions on long-term efficacy [30].

The WAVES randomized controlled trial which compared the Swank diet to the Wahls diet in 87 RRMS patients over 24 weeks found that the Swank diet participants experienced significant fatigue reduction (FSS scores decreased by –1.01 ± 0.24, *p* < 0.0001) and a 9.25-point improvement in physical QoL (*p* < 0.01) [31]. However, the Wahls group demonstrated even greater improvements in fatigue scores, suggesting that while dietary modification itself may be beneficial, the specific macronutrient composition could influence symptom outcomes [31]. The trial’s RCT design and parallel-arm methodology strengthened its validity; however, its short follow-up and reliance on self-reports limited insights into long-term outcomes. A secondary analysis of the WAVES trial examined cognitive outcomes in 77 RRMS patients over 36 weeks [32]. Swank diet adherence was associated with improved cognitive processing speed (SDMT-O scores increased from 57.8 ± 1.61 to 62.9 ± 1.60, *p* ≤ 0.05) and reduced perceived cognitive dysfunction (*p* ≤ 0.0002) [32]. However, cod liver oil supplementation was reported in some participants and, given that omega-3 fatty acids are known to enhance cognitive function, it remains unclear whether these cognitive benefits resulted from the Swank diet alone or from concurrent supplementation [32]. The study did not control for this confounder, making it difficult to determine whether improvements were attributable to the Swank diet alone. A 36-week randomized trial examined micronutrient intake among Swank diet adherents. Results showed significant reductions in inadequate intake of vitamin C (44.6–19.8%, *p* < 0.01), vitamin D (96.3–82.3%, *p* < 0.01), and vitamin E (50.1–32.3%, *p* = 0.03) [33]. Despite these improvements, deficiencies in calcium, magnesium, and zinc persisted, underscoring the need for supplementation [33]. Compared to the Wahls diet, Swank dieters had better calcium intake but continued deficiencies in magnesium and zinc. Given the essential role of these micronutrients in neuroprotection and immune regulation, their persistent deficiency raises concerns about whether long-term adherence to the Swank diet may involuntarily contribute to suboptimal neurological health [33].

Overall, the Swank diet shows potential for reducing fatigue, enhancing cognitive function, and improving QoL in MS. However, limitations such as small sample sizes, reliance on self-reported data, and the lack of long-term follow-up reduce confidence in its efficacy. Additionally, the historical dominance of R.L. Swank’s research in this area raises concerns about bias and reproducibility. Future studies should explore whether modifications to its fat intake recommendations could optimize its benefits while minimizing potential micronutrient deficiencies.

### 4.4 Wahls diet

The Wahls diet, also known as the Modified Paleolithic Diet Intervention (MPDI), emphasizes micronutrient-dense foods, including nine or more cups of vegetables daily (leafy greens, sulfur-rich vegetables, and deeply colored produce), alongside lean proteins, nuts, and healthy fats. It eliminates gluten, dairy, and processed foods, with the aim of reducing inflammation and supporting mitochondrial function [31]. The diet’s proposed benefits stem from its high intake of antioxidants, omega-3 fatty acids, and essential vitamins, which may modulate neuroinflammation and oxidative stress.

Irish et al. executed an RCT investigating the effects of the MPDI in 34 patients with RRMS (MPDI: *n* = 17; control: *n* = 17) [34]. The MPDI group showed a 16.2% improvement in mental health-related QoL (*p* = 0.02) and a significant reduction in fatigue (*p* = 0.03). Additionally, vitamin K levels increased by 262% (*p* = 0.02), which may have neuroprotective implications, though its direct impact on MS remains unclear [34]. In contrast, the control group experienced declines in fatigue and QoL scores. However, the small sample size limits the generalizability of these findings [34]. A 12-month single-arm study on 10 SPMS patients was conducted using a multimodal intervention (MPDI, dietary supplements, progressive exercise, and neuromuscular electrical stimulation) [35]. Results showed a significant decrease in FSS scores from 5.7 to 3.32 (*p* = 0.0008). A follow-up study involving 20 MS patients confirmed similar reductions in fatigue and improvements in QoL (*p* < 0.0005) [36]. However, since both studies lacked control groups and combined multiple interventions, it remains unclear how much of the benefit was due to diet alone. In the Wahls Behavioral Approach to MS With Diet & Exercise Study (WAVES) trial, Wahls dieters had higher intakes of vitamin A (13.1%, *p* < 0.01), vitamin C (1.5%, *p* < 0.01), vit amin D (86.6%, *p* < 0.01), vitamin E (26.7%, *p* < 0.01), and magnesium (28.5%, *p* < 0.01). However, despite supplementation, calcium (86.5%, *p* < 0.01), thiamin (13.6%, *p* = 0.03), and vitamin B12 (17.4%, *p* < 0.01) deficiencies persisted [33]. The restrictive nature of the Wahls diet, particularly the exclusion of grains and dairy, may explain the observed B12 deficiency [33]. This raises concerns about potential nutritional risks for long-term adherence. The secondary analysis of the WAVES trial [32] found an association between improved serum fatty acid profiles and better cognitive function among Wahls diet adherents. However, whether these changes translate into long-term neuroprotection remains uncertain.

The Wahls diet shows promise in improving MS symptoms, particularly fatigue and QoL. However, small sample sizes and short trial durations limit conclusions on its long-term efficacy. Additionally, studies where supplementation was included to address potential nutrient deficiencies caused by the restrictive nature of the diet may have positively influenced the results. This highlights the need to account for confounding variables to ensure accurate findings [37]. Larger, long-term studies are essential to determine its sustainability, safety, and role in MS management.

### 4.5 Ketogenic diet

Preclinical studies, including *in vitro* and animal models, suggest that the ketogenic, or keto, diet may have neuroprotective and anti-inflammatory effects in MS. While these findings are promising, their translation to human populations remains under investigation. A narrative review [38] found that the diet increases brain-derived neurotrophic factor, which supports myelin regeneration and neuronal repair. Their findings also indicated improvements in physical function, reduced fatigue, and lower neurofilament light chain levels, a biomarker of nerve damage. The ketogenic diet also decreased proinflammatory cytokine levels and inflammation markers, which are associated with improved QoL. Koh et al. [39] demonstrated that in an experimental autoimmune encephalomyelitis (EAE) murine model of MS, the ketogenic diet improved memory and mobility. MRI scans also revealed reduced brain atrophy in ketogenic diet-fed mice compared to controls.

In terms of immune modulation, Di Majo et al. study reported that ketone bodies lower nucleotide-binding domain, leucine-rich–containing family, pyrin domain–containing-3 inflammasome activity, a key driver of inflammation in autoimmune diseases [40]. Additionally, the ketogenic diet increases adenosine levels, an anti-inflammatory molecule that helps regulate immune activation. A 6-month clinical study of 65 patients with relapsing MS found the ketogenic diet to be safe and associated with significant improvements in physical (67 ± 16 vs 79 ± 12, *p* < 0.001) and mental QoL (71 ± 17 vs 82 ± 11, *p* < 0.001) [41]. Additionally, 50% of participants reported reduced fatigue and depression scores. Objective measures also improved, with significant gains in Expanded Disability Status Scale (EDSS) scores (2.3 ± 0.9 vs 1.9 ± 1.1, *p* < 0.001), walking distance (6-min walk: 1,631 ± 302 vs 1,733 ± 330 ft, *p* < 0.001), and hand dexterity (Nine-Hole Peg Test: 21.5 ± 3.6 vs 20.3 ± 3.7 s, *p* < 0.001) [41]. Despite these promising results, long-term adherence to the ketogenic diet remains challenging due to restrictive dietary requirements and side effects. One study noted that the ketogenic diet may disrupt lipid profiles and increase the risk of nutritional deficiencies [9]. Similarly, another study identified common adverse effects, including constipation, nausea, vomiting, and reduced appetite [42].

Although the ketogenic diet shows promise in reducing fatigue, depression, and inflammation in MS, evidence remains mixed, particularly in human studies. Large-scale, long-term trials are essential to determine its clinical viability, long-term safety, and integration into MS treatment protocols.

### 4.6 IF and calorie restriction

IF is being explored as a complementary approach in MS management, with promising findings from experimental studies and early clinical trials suggesting potential benefits in inflammation and neuroprotection. IF regimens fall into three main types: (1) time-restricted eating (TRE), which limits daily caloric intake to a fixed timeframe (e.g., 8-h eating window); (2) alternate-day fasting, which alternates between fasting and feasting days; and (3) the 5:2 method, involving five regular eating days and two non-consecutive fasting days per week [43,44,45]. An experimental study from 2016 [46] found that initiating IF during EAE’s prodromal stage reduced disease severity, promoted spinal cord remyelination, and decreased proinflammatory cytokines (IFN-γ, TNF-α), while increasing anti-inflammatory IL-10 levels. These findings suggest IF may offer neuroprotective and anti-inflammatory benefits in early MS-like disease models. Complementary studies have also demonstrated clinical improvement and increased myelination in EAE models following IF [47]. The gut microbiota is increasingly recognized for its role in systemic inflammation and metabolic regulation [48]. Leptin levels, which correlate with inflammatory responses, were negatively associated with gut microbiome diversity in both IF and ad libitum-fed groups (*r* = −0.51, *p* < 0.005), consistent with prior research [49]. These findings suggest that IF-induced microbiome changes may play a role in modulating MS-related inflammation and metabolism.

In clinical settings, preliminary investigations suggest IF may help in weight management and provide psychological benefits for individuals with MS [50]. TRE has been investigated in adults with MS, with early findings suggesting potential feasibility and metabolic benefits. A randomized controlled trial [51] explored IF’s short-term effects during MS relapses, highlighting metabolic shifts and gut microbiome alterations as key mechanisms. These findings underscore the need for systematic reviews to indicate IF’s therapeutic potential in MS management.

Fasting-mimicking diet cycles have shown beneficial effects on immune and endocrine functions in both animal models and humans. However, the long-term feasibility of IF in MS remains unclear, as restrictive fasting regimens may pose risks such as fatigue, headaches, and potential nutrient deficiencies. In MS, prolonged fasting could also impact bone mineral density and menstrual health, particularly in women [37].

Further research is needed to determine the safety, practicality, and optimal fasting protocols for MS management, ensuring that potential benefits outweigh any associated risks.

### 4.7 Gluten-free diet

The gluten-free diet eliminates all forms of gluten, a protein found in wheat, rye, and barley, and is commonly followed by individuals with celiac disease or gluten sensitivity. In MS, emerging research suggests that gluten may contribute to inflammation and immune dysregulation, prompting investigations into its potential role in disease activity. Gluten has been hypothesized to contribute to MS pathophysiology by increasing intestinal permeability, leading to systemic immune activation. This may compromise blood-brain barrier integrity, allowing autoreactive T cells to infiltrate the CNS and cross-react with nerve proteins, potentially exacerbating neuroinflammation [52].

A retrospective, cross-sectional study including 186 MS patients examined the impact of gluten and dairy consumption on disease activity [53]. Dietary intake was assessed and correlated with MRI lesion load and patient-reported symptoms. No significant differences in lesion progression were observed between gluten-consuming and gluten-free groups (*p* = 0.28) [53]. However, a subset of gluten-sensitive individuals experienced reductions in gastrointestinal symptoms and fatigue upon adopting a gluten-free diet. The study’s reliance on self-reported dietary intake introduces recall bias, and its observational nature precludes causal conclusions [53]. Nevertheless, the use of No Evidence of Disease Activity strengthens the findings by providing an objective measure of MS disease activity. In 2023, Zevallos et al. carried out an experimental study to investigate the effects of wheat amylase-trypsin inhibitors (ATIs) – compounds found in gluten-containing foods – on CNS inflammation in an EAE mouse model of MS [54]. Mice fed an ATI-rich diet exhibited significantly elevated proinflammatory cytokine levels, including IL-17 and TNF-α (*p* < 0.01), along with worsened CNS inflammation compared to controls. These findings suggest that gluten-derived ATIs may contribute to neuroinflammation in MS [54]. However, as an animal study, direct clinical translation is limited, necessitating further investigation in human populations.

### 4.8 UPFs

UPFs have been implicated in MS progression, with evidence suggesting their role in promoting chronic inflammation, altering gut microbiota, and impairing immune regulation. A cross-sectional study analyzed dietary data from 508 healthy controls and 267 adults with CNS demyelination using a food frequency questionnaire [55]. After adjusting for age, sex, smoking, BMI, and physical activity, each additional daily serving of UPFs was associated with an 8% higher risk of CNS demyelination. While further research is needed to confirm causal mechanisms, UPFs’ high content of refined sugars, unhealthy fats, and synthetic additives has been linked to neuroinflammation and immune dysregulation in MS [55]. Another cross-sectional study examined the link between UPF consumption and MS severity, assessing dietary intake via a food frequency questionnaire and disease progression using validated disability scores (EDSS and Multiple Sclerosis Severity Score) [56]. Results showed that individuals with higher UPF intake had a nearly threefold increased likelihood of moderate-to-high MS severity (OR = 2.97, 95% confidence interval [CI]: 1.13–7.77, *p* < 0.05). Before adjusting for confounders, the odds were 2.28 times higher (95% CI: 1.04–5.01) [56]. After controlling for confounding factors, including age, BMI, and smoking, the association remained significant (OR = 2.46, 95% CI: 1.04–5.83), reinforcing the potential link between UPF consumption and MS severity. These findings suggest a potential role of UPFs in worsening MS severity, though further studies are needed to confirm causality and underlying mechanisms [56].

Excess salt intake, a common feature of many UPFs, has also been linked to immune dysregulation in MS. Results from an experimental study demonstrated that high sodium intake promotes the differentiation of proinflammatory TH17 cells, which are implicated in MS pathogenesis and contribute to neuroinflammation [57]. This effect was mediated by activation of the p38/MAPK signaling pathway via critical regulators of immune cell function. Blocking these pathways prevented the salt-induced increase in TH17 cells, suggesting that dietary salt may contribute to MS pathogenesis by promoting neuroinflammation [57].

While studies indicate a potential link between UPF consumption and MS severity, most evidence is observational, limiting causal interpretations. Given the inflammatory and immune-modulating properties of UPFs, further research is needed to determine their long-term impact on MS progression and symptom management. In the meantime, reducing UPF consumption in favor of whole, minimally processed foods may be a beneficial strategy for individuals with MS.

## 5 Role of key nutrients and components

### 5.1 Vitamin D

Vitamin D plays a key role in immune modulation and has been extensively studied for its potential impact on MS. It regulates immunity by promoting anti-inflammatory responses and inhibiting autoreactive lymphocyte activation, reducing immune dysfunction. Additionally, vitamin D is involved in the regulation of T cells and has been associated with HLA-DRB1*1501, a genetic variant strongly linked to an increased risk of MS [58]. After MS onset, vitamin D3 supplementation has been reported to enhance regulatory T cells while reducing pro-inflammatory TH1 and TH17 cells, resulting in decreased IL-17 levels and increased IL-10 levels [59].

A systematic review and meta-analysis of 14 case-control studies found that individuals with vitamin D levels below 50 nmol/L had a 54% higher risk of developing MS (OR 1.54; 95% CI 1.05, 2.24) [60]. Similarly, a randomized, double-blind, controlled trial of Alfacalcidol (1α-hydroxy vitamin D3) showed promising results in reducing fatigue and relapse rates, with Alfacalcidol users experiencing a 42% decrease in fatigue (*p* = 0.007) and a lower relapse rate (*p* = 0.006) [61]. Furthermore, an umbrella review assessing systematic reviews and meta-analyses involving over 39,000 participants suggested that individuals with specific vitamin D-related gene polymorphisms had an 8% reduced risk of developing MS (OR 0.92; 95% CI 0.86, 0.98; *I*² = 0%, *p* > 0.99) [62].

A longitudinal study of 73 RRMS patients indicated that higher vitamin D levels (>100 nmol/L) were associated with reduced exacerbation risk, with a 30% lower risk for those with moderate vitamin D levels and a 50% lower risk for those with high levels compared to the low-level group [63]. Likewise, a prospective cohort study of 109 Chinese patients with a median follow-up of one year found that lower serum vitamin D levels were significantly associated with higher exacerbation risk (*p* < 0.0001) and worse EDSS scores (*p* = 0.010) [64]. Interestingly, some studies suggest a bidirectional relationship or reverse causality, where MS itself may contribute to lower vitamin D levels rather than vice versa [64]. A systematic review analyzing studies on vitamin D published between 2005 and 2015 found potential benefits, including reduced exacerbation rates, fewer new brain lesions, and improved inflammatory response [4]. However, variability in vitamin D dosing and treatment duration across studies limited direct comparisons and prevented definitive conclusions. Additionally, some studies found no effect on bone loss prevention, which is particularly relevant given the increased risk of osteoporosis in MS patients. This suggests that vitamin D supplementation alone may not be sufficient to prevent bone loss, necessitating additional interventions, particularly for high-risk groups such as postmenopausal women and individuals on glucocorticoids, anticoagulants, or chemotherapy [4].

On the other hand, not all evidence supports the benefits of vitamin D supplementation. A systematic review of RCTs on high-dose vitamin D supplementation found no significant improvements in clinical outcomes, including EDSS (*p* = 0.91), annual relapse rates (ARR) (*p* = 0.26), or the occurrence of new T2 lesions (*p* = 0.08) [65]. Similarly, a meta-analysis found no significant association between high-dose vitamin D and relapse risk (OR 0.98; 95% CI 0.45–2.16) [66]. Additionally, a study conducted by Mehrabadi and Zahedi on inflammatory markers in MS patients found that vitamin D supplementation did not significantly affect levels of IL-10, IL-17, or IFN-γ [67].

These mixed findings underscore the need for further research to clarify the role of vitamin D in MS. Larger, well-controlled RCTs with longer follow-up periods and standardized dosing protocols are necessary to determine its true impact on disease progression and clinical outcomes.

### 5.2 Omega-3 fatty acids

Current literature on the role of omega-3 fatty acids in managing MS highlights promising but inconsistent results, reflecting variability in study designs, outcomes, and patient populations. Omega-3 fatty acids, particularly eicosapentaenoic acid and docosahexaenoic acid, are hypothesized to exert neuroprotective and anti-inflammatory effects, yet their efficacy in MS remains debated. DHA constitutes over 90% of omega-3 polyunsaturated fatty acids in the brain and 20% of total brain lipids. It plays a key role in neuronal membrane integrity, synaptic function, and neuroprotection by modulating membrane fluidity and gene expression [68]. While these mechanisms suggest a potential role in MS, further research is needed to determine its specific impact on disease progression.

A six-month randomized controlled trial conducted by AlAmmar et al. involving 100 RRMS patients found a 15% reduction in relapse frequency (*p* < 0.05) and a 10% improvement in fatigue scores (*p* = 0.03), but no significant changes were detected in MRI lesion activity (*p* = 0.6) [69]. These findings suggest that omega-3 supplementation may help alleviate fatigue but does not significantly impact MRI-detected neurological changes [69]. Similarly, a longitudinal cohort study was performed over a period of 12 months with 150 RRMS patients and observed a 12% reduction in IL-6 levels (*p* = 0.04), indicating mild anti-inflammatory effects [29]. However, the study reported no significant changes in relapse rates (*p* = 0.5) or cognitive outcomes (*p* = 0.7), emphasizing the limited clinical utility of omega-3s over the long term.

In contrast, a prospective cohort study explored omega-3’s preventative potential in a prospective cohort study of 282 participants at risk for MS [11]. The study found that higher omega-3 intake was associated with a 35% reduced risk of CNS demyelination (*p* = 0.01), particularly in individuals with adequate vitamin D levels. However, as an observational study, it cannot establish causation, and further interventional studies are needed [11]. Another study conducted a scoping review on omega-3 supplementation in MS, reporting mixed findings [70]. Some studies showed slight reductions in inflammation and fatigue, while others found no impact on relapse rates or MRI lesion progression [70]. The inconsistency in results was attributed to variations in study designs, omega-3 dosages, and patient populations. Overall, these findings underscore the need for more standardized, long-term trials to clarify omega-3’s precise role in MS prevention and symptom management [70]. Similarly, in an observational study assessing high-dose biotin as a potential therapy for MS, 43 patients with different types of MS were administered 300 mg/day of biotin daily [71]. No improvements were evident. In fact, over one-third of patients experienced deterioration in their neurological status, with the most common symptoms being worsening lower limb weakness, gait instability, and heightened lower limb spasticity [71]. One major limitation of this study is the variation in biotin dosing. Additionally, the sample consisted of older patients in a more progressive disease state with slightly higher EDSS scores compared to previous studies. However, the study demonstrated a higher compliance rate due to the single-dose administration. Furthermore, the diversity of the sample improved the generalizability of the results [71].

The findings of Torkildsen et al., double-blind RCT, revealed no significant effect of omega-3 supplementation on MRI lesion activity, relapse rates, disability progression, or fatigue over 24 months [72]. Although these findings suggest limited clinical benefit, the study’s small sample size may have reduced its ability to detect subtle effects [69].

The efficacy of omega-3 fatty acids in MS remains inconclusive, with mixed findings on their impact on inflammation, fatigue, and disease progression. While some studies reported slight reductions in inflammation and fatigue, others found no significant effect on relapse rates or MRI lesion progression. These discrepancies may be due to variations in omega-3 dosage, study design, and patient characteristics. Future research should prioritize large-scale, well-controlled RCTs with standardized dosing and long-term follow-up to clarify whether omega-3 supplementation provides meaningful clinical benefits for MS management.

### 5.3 Antioxidants

Antioxidant-rich diets are gaining recognition for their ability to mitigate oxidative stress, a key factor in MS progression. In a randomized controlled trial [73], a low-fat diet alone (control, *n* = 4) was compared to a low-fat diet with antioxidant supplementation from *Lipia citriadora*, a vegetal extract (200 mg, 5 times/week; intervention, *n* = 5). After 14 days, the supplemented group demonstrated significant reductions in CRP levels (−9.125 mg/L, *p* < 0.05) and serum IL-6 (−3.26, *p* < 0.05), as well as increased catalase activity (35.09 vs 1.28 in controls, *p* < 0.05), suggesting improved antioxidant defense. Urine isoprostane levels, a marker of lipid peroxidation, decreased (−1889.50 pg/mL) in the intervention group but rose in controls (+1634.66 pg/mL). While these results suggest potential anti-inflammatory and antioxidant benefits, the small sample size and short study duration limit their generalizability. Larger, longer-term studies are needed to confirm these findings.

A single-center randomized clinical trial investigated an antioxidant-rich, anti-inflammatory diet with symbiotics in progressive MS patients (*n* = 69) [74] (110.5 ± 75.9–44.7 ± 49.3 μg/g, *p* < 0.001). Additionally, vision capacity improved (*p* = 0.02), suggesting broader systemic effects of the intervention [74]. A single-center RCT investigated the effects of an antioxidant-rich, anti-inflammatory diet combined with symbiotic supplementation in patients with progressive MS (*n* = 69) [74]. Over 4 months, the intervention group demonstrated a significant reduction in gut inflammation, as evidenced by a decrease in fecal calprotectin levels from 110.5 ± 75.9 to 44.7 ± 49.3 μg/g (*p* < 0.001). This suggests that the diet may have modulated gut microbiota and reduced intestinal inflammation, which is increasingly recognized as a contributor to MS pathogenesis. Additionally, the intervention group showed improved vision capacity (*p* = 0.02), indicating potential systemic benefits beyond gut health. In a complementary study on the same subjects, another single-center, single-blind RCT reported reductions in fatigue (MFIS score from 51.0 ± 12.2 to 40.5 ± 11.9, *p* < 0.001), pain sensitivity (global pain scores from 92.9 ± 27.9 to 78.9 ± 22.0, *p* < 0.001), and minor improvements in bowel/bladder control (*p* = 0.041) [75]. While these results indicate potential symptom relief, the absence of a control group limits causal interpretations. Further research with larger, controlled trials is needed to confirm these effects.

An experimental study was carried out by Guan et al. in 2018 to evaluate the effects of vitamin E supplementation (400 mg/day for 3 months) in MS patients (*n* = 34) [76]. At baseline, MS patients exhibited elevated oxidative stress (urinary 8-iso-PGF2α: 62.5 ± 10.0 vs 47.0 ± 9.1 ng/mmol creatinine in controls, *p* < 0.001) and shorter telomere length (TL, 6.9 ± 1.0 vs 9.1 ± 1.4 kb, *p* = 0.027). After supplementation, urinary 8-iso-PGF2α levels decreased to 53.0 ± 9.6 ng/mmol (*p* < 0.001) in the treatment group, whereas oxidative stress markers worsened in the untreated group. TL remained stable in the supplemented group but declined in controls.

Despite its antioxidant effects, vitamin E supplementation did not lead to significant changes in clinical disability, as measured by EDSS. These findings suggest vitamin E may help mitigate oxidative stress and preserve telomere length. However, its lack of clinical improvements highlights the need for further research to assess its potential role in MS symptom management.

### 5.4 Polyphenols

**Figure 2:**
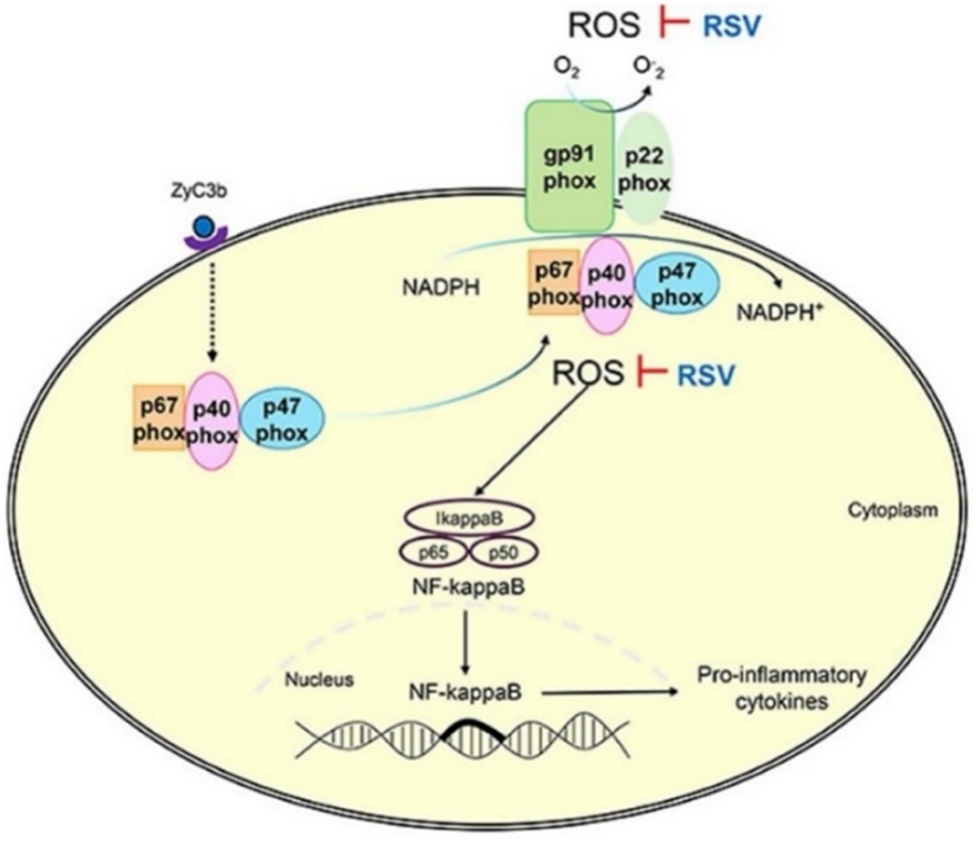
The role of RSVs in reducing ROS production, which may help mitigate inflammation and oxidative stress in MS patients [82].

Polyphenols, known for their antioxidant and anti-inflammatory properties, have been investigated for their role in MS management. These compounds, including flavonoids, RSV, catechins, and curcumin, help regulate inflammatory markers and may mitigate oxidative stress, a key factor in MS progression [77]. Curcumin, one of the most studied polyphenols, has demonstrated significant antioxidant effects in MS. For example, an RCT [78] reported a significant reduction in inflammatory markers, including FoxP3 expression (*p* = 0.0005) and TGF-β levels (*p* = 0.0005), after 6 months of nanocurcumin supplementation in RRMS patients. Another RCT showed a decrease in inflammatory mediators such as miR-145 (*p* < 0.0001) and NF-κB (*p* < 0.0001), highlighting its role in regulating immune responses [79]. However, a prospective RCT examining curcumin as an adjunct to interferon beta-1a (IFN β-1a) yielded mixed results. The curcumin-IFN group exhibited fewer contrast-enhancing lesions (*p* = 0.0167), but no significant effects were observed on clinical outcomes such as EDSS or relapse rate. High dropout rates, particularly at the one-year follow-up, may have influenced these findings [80]. RSV, a polyphenol primarily found in grapes and red wine, exerts neuroprotective effects by reducing ROS production via NADPH oxidase inhibition, thereby mitigating oxidative stress and inflammation [81], as shown in Figure 2.

A cross-sectional study found that RSV significantly reduced IL-6 levels by 49% in controls, but levels in MS patients remained unchanged, suggesting its anti-inflammatory effects may be more pronounced in non-diseased individuals or influenced by disease-related metabolic differences [82]. A meta-analysis of RCTs showed RSV supplementation significantly lowered TNF-α (*p* = 0.002) and hs-CRP (*p* = 0.033), indicating synergistic effects with pharmacological treatments [83]. Despite promising findings, inconsistent study methodologies and follow-up periods warrant further research to determine long-term effects.

In addition to RSV, epigallocatechin-3-gallate (EGCG), a polyphenol in green tea, has been studied for its anti-inflammatory effects. It downregulates chemokines and pro-inflammatory cytokines while inhibiting MAPK, STAT, and NF-κB signaling [84]. In a prospective double-blind RCT involving 122 RRMS patients, no significant differences were observed after 18 months of EGCG intervention in primary outcomes such as lesion number, lesion volume, or brain volume changes. Clinical outcomes, including EDSS and ARR, also showed no significant differences. While overall findings did not show a significant benefit, subgroup analysis suggested that EGCG may help stabilize disease activity in patients with a history of lower disease activity, warranting further investigation in targeted populations [85].

While polyphenols like curcumin, RSV, and EGCG show promise in reducing inflammation and oxidative stress in MS, clinical evidence remains inconsistent. Further large-scale, well-controlled trials are needed to determine their long-term efficacy and therapeutic potential.

### 5.5 Biotin

Several studies have reported that high-dose biotin supplementation may improve certain clinical symptoms in patients with MS, though findings remain inconsistent. In an open-label pilot study, 23 patients with primary or SPMS received biotin (100–300 mg/day) for an average of 9.2 months. While 91.3% of patients (21/23) demonstrated some level of improvement, the degree of benefit varied among different measures, including walking distance, EDSS scores, time to walk 25 feet, and visual acuity [86]. The proposed mechanism suggests that biotin enhances energy production in nerve cells and supports myelin repair by stimulating fatty acid synthesis [86]. A randomized, double-blind, placebo-controlled trial with 145 patients showed that after 12 months, 12.6% (13/103) of patients in the biotin (100 mg/day) group achieved a ≥1-point EDSS improvement, compared to 0% in the placebo group [87]. However, statistical significance was not reported for this outcome, limiting the strength of the findings. Despite this, the study suggests that MD1003 (biotin) may hold therapeutic potential by targeting neuron and oligodendrocyte metabolism.

In contrast, other studies have failed to demonstrate significant benefits of high-dose biotin in MS, with some reporting no improvement in clinical outcomes or suggesting possible adverse effects. A prospective study was conducted with 178 patients with progressive MS over 12 months and found no significant changes in disability scores, raising doubts about biotin’s clinical efficacy [88]. Similarly, an observational study including 43 patients with different types of MS who received 300 mg/day of biotin observed no clinical improvements [71]. Over one-third of patients experienced worsening neurological symptoms, including increased lower limb weakness, gait instability, and spasticity. However, the study could not establish whether symptom worsening was due to biotin itself or the natural progression of MS [71]. A major limitation was the variation in patient characteristics, as participants were older and in a more advanced disease stage than in previous studies. The study did, however, report high compliance rates due to the single-dose administration [71].

Overall, while some studies suggest that high-dose biotin may provide benefits for a subset of MS patients, others report no improvement or potential worsening of symptoms. These mixed results highlight the need for further research, particularly in identifying which MS subtypes, if any, may respond favorably to biotin therapy.

## 6 Microbiome

The gut–brain axis, mediated by the gut microbiome, influences immune regulation in MS. Gut bacteria produce SCFAs like butyrate, which help maintain gut barrier integrity and regulate T-regulatory cells, key mediators of immune tolerance. SCFAs also reduce the production of pro-inflammatory cytokines, which contribute to autoimmune activity in MS [89]. Dysbiosis, an imbalance in gut microbiota, has been linked to increased intestinal permeability and heightened systemic inflammation, both of which are associated with MS progression [90]. Pathogenic gut bacteria, including *Akkermansia* and *Prevotella* species, have been implicated in molecular mimicry, where microbial antigens resemble myelin proteins, triggering autoreactive T cells that attack the CNS and worsen MS symptoms [91]. A study of 18 RRMS cases and 17 controls identified significant microbial imbalances in MS patients, including increased Desulfovibrionaceae and Christensenellaceae and decreased Lachnospiraceae and Ruminococcaceae (*p* and *q* < 0.000005). These shifts were linked to changes in glutathione metabolism, suggesting a role for oxidative stress (*p* = 0.017). Additionally, beta diversity differences were observed based on immunomodulatory drug exposure (*p* < 0.02), indicating that MS treatments may influence gut microbiota [92]. Dietary interventions, including prebiotics and probiotics, have shown potential in modulating gut microbiota and reducing MS-related inflammation. Prebiotics, such as non-digestible oligosaccharides found in dairy, promote beneficial bacteria like Bifidobacteria, which enhance immune function [93]. Probiotics, including Lactobacillus species, strengthen the gut barrier and reduce systemic inflammation, potentially mitigating MS progression [94]. Probiotics also regulate immune responses by increasing IL-10 production, an anti-inflammatory cytokine that plays a key role in reducing CNS inflammation and supporting immune tolerance in MS [95]. Omega-3 fatty acids and polyphenols contribute to gut-brain axis regulation by enhancing microbial diversity and lowering inflammatory markers. These effects may help mitigate neuroinflammation and slow MS progression [96].

## 7 Limitations and controversies

Dietary interventions in MS management show potential, but significant limitations prevent drawing definitive conclusions from current research. A key limitation is inconsistency in study design – including small sample sizes, short follow-up periods, and high dropout rates – which reduces reliability and generalizability. These factors hinder assessments of long-term effects on disease progression and symptom management. Furthermore, many studies rely on subjective outcome measures, such as self-reported fatigue, pain, and QoL, which introduce recall bias and reduce applicability to broader MS populations.

While certain dietary patterns and nutrients show potential benefits, the evidence remains inconsistent. Omega-3 fatty acids, antioxidants, and vitamin D have been linked to improved inflammation and immune function, but their effects vary depending on study design, disease subtype, medication use, genetic predisposition, and comorbidities, all of which influence dietary response. Additionally, most studies on the Swank and Wahls diets have been conducted by their inventors’ research groups, raising concerns about bias. Many dietary interventions, including the Wahls, Swank, and ketogenic diets, have only been studied for short durations (often four months or less), limiting insight into their long-term sustainability, safety, and efficacy.

Moreover, highly restrictive diets such as the Wahls, Swank, and ketogenic diets, pose significant adherence challenges. Long-term compliance is difficult due to dietary complexity, cost, and social limitations. Additionally, improper implementation may lead to nutritional deficiencies, necessitating further guidance from healthcare professionals. The complex interplay between diet, gut microbiota, and immune regulation further complicates the implementation of long-term dietary strategies in MS. Another limitation of this review is the inclusion of Google Scholar, which primarily indexes grey literature rather than exclusively peer-reviewed scientific databases. This should be considered when interpreting the findings.

While dietary strategies hold promises as complementary therapies for MS, the current body of evidence remains inconclusive. Future research should prioritize large-scale RCTs with extended follow-up periods, larger sample sizes, and objective biomarkers (e.g., MRI lesion activity and cytokine levels) to better assess the long-term effects of dietary interventions in MS management.

## 8 Conclusion

This review highlights the potential of dietary interventions as complementary strategies in MS management. Given the complexity of MS, effective treatment should address inflammation, neurodegeneration, and oxidative stress.

Evidence suggests that Mediterranean and plant-based diets may reduce fatigue, improve QoL, and modulate inflammatory markers. Restrictive diets such as ketogenic, Swank, and Wahls show promise but pose adherence challenges due to side effects and nutritional deficiencies. Similarly, IF and calorie restriction may offer benefits but require further investigation. Key nutrients like omega-3 fatty acids, antioxidants, vitamin D, and probiotics play roles in immune regulation and neuroprotection, though inconsistencies in study designs limit definitive conclusions.

Personalized dietary interventions, tailored to disease stage and comorbidities, could enhance MS management. While diet alone cannot replace conventional therapies, integrating evidence-based nutritional strategies into a comprehensive treatment plan may improve long-term patient outcomes and QoL. Large-scale randomized trials are essential to establish clear dietary guidelines for MS care.

## Supplementary Material

Supplementary Table
