# Supplementary material for: From prevention to management: Exploring the impact of diet on multiple sclerosis
Source: Transl Neurosci. 2025 May 14;16(1):20250371. doi: 10.1515/tnsci-2025-0371 (PMC12086631; doi:10.1515/tnsci-2025-0371)
Supplement: Supplementary Table [file tnsci-2025-0371-sm.pdf]

Supplementary material

Table S1: Summary of findings: diet and multiple sclerosis

Intervention	Key findings	Study designs	Recommendations	References
Mediterranean diet	Associated with reduced fatigue, improved QoL, and modulation of inflammatory markers. Case–control study linked higher adherence to lower MS risk	RCTs, observational studies	Large-scale RCTs with standardized dietary protocols are needed	[6–11]
Plant-based diet	Observational studies suggest lower relapse rates and improved QoL. Some evidence supports reduced inflammation, but RCTs remain limited	RCTs, systematic reviews	Future trials should evaluate long-term adherence and nutrient adequacy	[12–15]
Swank diet	RCTs report fatigue reduction and QoL improvements. Cognitive function may improve, but concerns exist over long-term micronutrient deficiencies	RCTs, observational studies	Modify fat intake recommendations to improve sustainability and nutrient balance	[16–19]
Wahls diet	Shows promise in reducing fatigue, improving QoL, and enhancing micronutrient intake. Higher vitamin and antioxidant levels than Swank diet	RCTs, pilot studies	Investigate long-term adherence strategies and whether supplementation can mitigate potential deficiencies	[17,20–22]
Ketogenic diet	May reduce inflammation and promote immune regulation, but human studies are limited	Animal models, limited clinical data	Long-term trials needed to assess metabolic and neurological effects	[2,23–28]
IF	Some studies report neuroprotective effects and reduced fatigue. Potential benefits for energy metabolism and brain function	RCTs, reviews	Long-term trials needed to assess metabolic and neurological effects	[29–34]
Gluten-free diet	Results are inconclusive; some studies suggest symptom improvement, but no strong clinical support	Observational studies, preclinical studies,	More controlled trials needed to confirm gluten-free diet-MS relationships	[35–37]

(*Continued*)

Table S1: *Continued*

Intervention	Key findings	Study designs	Recommendations	References
	May benefit those with gluten sensitivity			
UPFs and high-SFA diets	Associated with increased inflammation, gut dysbiosis, and worsening MS symptoms	Observational studies, meta-analyses	More controlled trials needed to confirm diet-inflammation-MS relationships	[3,5,38–40]
Vitamin D	Low vitamin D levels linked to increased MS risk and disease activity. Supplementation may reduce relapse rates and modulate immune function	RCTs, systematic revies	Determine optimal dosage and duration for MS management	[1,38–43]
Omega-3 fatty acids	Results are mixed; some studies show reduced inflammation and fatigue, while others find no impact on relapse rates or MRI lesions	RCTs, preclinical studies	Standardized dosing and long-term trials needed to clarify therapeutic potential	[4,44–48]
Antioxidants	May reduce oxidative stress and inflammation in MS. Vitamin C and E and other antioxidants have been studied for neuroprotection	RCTs, meta-analyses	More controlled trials needed to assess efficacy and safety in MS patients	[49–52]
Polyphenols	Curcumin, RSV, and green tea catechins have demonstrated anti-inflammatory and neuroprotective properties	RCTs, pilot studies	Develop optimized formulations with improved absorption	[53–61]
Biotin	High-dose biotin may promote myelin repair and improve clinical disability in progressive MS. Some trials report improved EDSS scores	RCTs, observational studies	Further research needed to confirm safety and efficacy	[47,62–64]
Microbiome	Gut microbiota composition influences inflammation and immune responses in MS. Probiotics and prebiotics may modulate gut health	Observational studies, meta-analyses	Investigate personalized microbiome-targeted interventions	[65–71]

References

[1] Bagur MJ, Murcia MA, Jiménez-Monreal AM, Tur JA, Bibiloni MM, Alonso GL, et al. Influence of diet in multiple sclerosis: A systematic review. Adv Nutr. 2017;8(3):463–72.

[2] Harirchian MH, Karimi E, Bitarafan S. Diet and disease-related outcomes in multiple sclerosis: A systematic review of clinical trials. Curr J Neurol. 2022;21(1):52–63.

[3] Hoover DB. Cholinergic modulation of the immune system presents new approaches for treating inflammation. Pharmacol Ther. 2017;179:1–16.

[4] Hoare S, Lithander F, van der Mei I, Ponsonby AL, Lucas R. Higher intake of omega-3 polyunsaturated fatty acids is associated with a decreased risk of a first clinical diagnosis of central nervous system demyelination: Results from the Ausimmune Study. Mult Scler. 2016;22(7):884–92.

[5] Riccio P, Rossano R. Diet, gut microbiota, and vitamins D + A in multiple sclerosis. Neurotherapeutics. 2018;15(1):75–91.

[6] Skovgaard L, Trénel P, Westergaard K, Knudsen AK. Dietary patterns and their associations with symptom levels among people with multiple sclerosis: A real-world digital study. Neurol Ther. 2023;12(4):1335–57.

[7] Razeghi-Jahromi S, Doosti R, Ghorbani Z, Saeedi R, Abolhasani M, Akbari N, et al. A randomized controlled trial investigating the effects of a mediterranean-like diet in patients with multiple sclerosis-associated cognitive impairments and fatigue. Curr J Neurol. 2020;19(3):112–21.

[8] Giesser BS, Rapozo M, Glatt R, Patis C, Panos S, Merrill DA, et al. Lifestyle intervention improves cognition and quality of life in persons with early multiple sclerosis. Mult Scler Relat Disord. 2024;91:105897.

[9] Bagheri S, Soltani S, Moravejolahkami AR, et al. A randomized controlled trial investigating the effects of a Mediterranean-like diet in patients with multiple sclerosis-associated cognitive impairments and fatigue. Clin Nutr. 2021;40(9):4586–94. doi: 10.1016/j.clnu.2021.06.002.

[10] Snetselaar LG, Cheek JJ, Fox SS, Healy HS, Schweizer ML, Bao W, et al. Efficacy of diet on fatigue and quality of life in multiple sclerosis: A systematic review and network meta-analysis of randomized trials. Neurology. 2023;100(4):e357–66.

[11] Mirza AI, Zhu F, Knox N, Black LJ, Daly A, Bonner C, et al. Mediterranean diet and associations with the gut microbiota and pediatric-onset multiple sclerosis using trivariate analysis. Commun Med (Lond). 2024;4(1):148.

[12] Mohan M, Okeoma CM, Sestak K. Dietary gluten and neurodegeneration: A case for preclinical studies. Int J Mol Sci. 2020;21(15).

[13] Thirion F, Sellebjerg F, Fan Y, Lyu L, Hansen TH, Pons N, et al. The gut microbiota in multiple sclerosis varies with disease activity. Genome Med. 2023;15(1):1.

[14] Yadav V, Marracci G, Kim E, Spain R, Cameron M, Overs S, et al. Low-fat, plant-based diet in multiple sclerosis: A randomized controlled trial. Mult Scler Relat Disord. 2016;9:80–90.

[15] Walters SJ, Brazier JE. Comparison of the minimally important difference for two health state utility measures: EQ-5D and SF-6D. Qual Life Res. 2005;14(6):1523–32.

[16] Yu M, Jelinek G, Simpson-Yap S, Neate S, Nag N. Self-reported ongoing adherence to diet is associated with lower depression, fatigue, and disability, in people with multiple sclerosis. Front Nutr. 2023;10:979380.

[17] Wahls TL, Titcomb TJ, Bisht B, Eyck PT, Rubenstein LM, Carr LJ, et al. Impact of the Swank and Wahls elimination dietary interventions on fatigue and quality of life in relapsing-remitting multiple sclerosis: The WAVES randomized parallel-arm clinical trial. Mult Scler J Exp Transl Clin. 2021;7(3):20552173211035399.

[18] Titcomb TJ, Brooks L, Smith KL, Ten Eyck P, Rubenstein LM, Wahls TL, et al. Association between improved serum fatty acid profiles and cognitive function during a dietary intervention trial in relapsing-remitting multiple sclerosis. Nutr Neurosci. 2023;26(10):741–51. doi: 10.1080/1028415X.2021.2002457.

[19] Titcomb TJ, Brooks L, Smith KL, Ten Eyck P, Rubenstein LM, Wahls TL, et al. Change in micronutrient intake among people with relapsing-remitting multiple sclerosis adapting the Swank and Wahls diets: An analysis of weighed food records. Nutrients. 2021;13(10):3507.

[20] Irish AK, Erickson CM, Wahls TL, Snetselaar LG, Darling WG. Randomized control trial evaluation of a modified Paleolithic dietary intervention in the treatment of relapsing-remitting multiple sclerosis: A pilot study. Degener Neurol Neuromuscul Dis. 2017;7:1–18.

[21] Bisht B, Darling WG, Grossmann RE, Shivapour ET, Lutgendorf SK, Snetselaar LG, et al. A multimodal intervention for patients with secondary progressive multiple sclerosis: Feasibility and effect on fatigue. J Altern Complement Med. 2014;20(5):347–55.

[22] Bisht B, Darling WG, Shivapour ET, Lutgendorf SK, Snetselaar LG, Chenard CA, et al. Multimodal intervention improves fatigue and quality of life in subjects with progressive multiple sclerosis: A pilot study. Degener Neurol Neuromuscul Dis. 2015;5:19–35.

[23] Atabilen B, Akdevelioğlu Y. Effects of different dietary interventions in multiple sclerosis: A systematic review of evidence from 2018 to 2022. Nutr Neurosci. 2023;26(12):1279–91.

[24] Dyńka D, Kowalcze K, Paziewska A. The role of ketogenic diet in the treatment of neurological diseases. Nutrients. 2022;14(23).

[25] Koh S, Dupuis N, Auvin S. Ketogenic diet and neuroinflammation. Epilepsy Res. 2020;167:106454.

[26] Di Majo D, Cacciabaudo F, Accardi G, Gambino G, Giglia G, Ferraro G, et al. Ketogenic and modified mediterranean diet as a tool to counteract neuroinflammation in multiple sclerosis: Nutritional suggestions. Nutrients. 2022;14(12).

[27] Brenton JN, Lehner-Gulotta D, Woolbright E, Banwell B, Bergqvist AGC, Chen S, et al. Phase II study of ketogenic diets in relapsing multiple sclerosis: Safety, tolerability and potential clinical benefits. J Neurol Neurosurg Psychiatry. 2022;93(6):637–44.

[28] Ortí JER, Cuerda-Ballester M, Sanchis-Sanchis CE, Lajara Romance JM, Navarro-Illana E, García Pardo MP. Exploring the impact of ketogenic diet on multiple sclerosis: Obesity, anxiety, depression, and the glutamate system. Front Nutr. 2023;10:1227431.

[29] Razeghi Jahromi S, Ghaemi A, Alizadeh A, Sabetghadam F, Moradi Tabriz H, Togha M. Effects of intermittent fasting on experimental autoimune encephalomyelitis in C57BL/6 mice. Iran J Allergy Asthma Immunol. 2016;15(3):212–9.

[30] Fitzgerald KC, Bhargava P, Smith MD, Vizthum D, Henry-Barron B, Kornberg MD, et al. Intermittent calorie restriction alters T cell subsets and metabolic markers in people with multiple sclerosis. EBioMedicine. 2022;82:104124.

[31] Sonnenburg JL, Bäckhed F. Diet-microbiota interactions as moderators of human metabolism. Nature. 2016;535(7610):56–64.

[32] Le Chatelier E, Nielsen T, Qin J, Prifti E, Hildebrand F, Falony G, et al. Richness of human gut microbiome correlates with metabolic markers. Nature. 2013;500(7464):541–6.

[33] Fitzgerald KC, Vizthum D, Henry-Barron B, Schweitzer A, Cassard SD, Kossoff E, et al. Effect of intermittent vs daily calorie restriction on changes in weight and patient-reported outcomes in people with multiple sclerosis. Mult Scler Relat Disord. 2018;23:33–9.

[34] Cignarella F, Cantoni C, Ghezzi L, Salter A, Dorsett Y, Chen L, et al. Intermittent fasting confers protection in CNS autoimmunity by altering the gut microbiota. Cell Metab. 2018;27(6):1222–35.e6.

[35] Zielińska M, Michońska I. Effectiveness of various diet patterns among patients with multiple sclerosis. Postep Psychiatr Neurol. 2023;32(1):49–58.

[36] Temperley IA, Seldon AN, Reckord MA, Yarad CA, Islam FT, Duncanson K, et al. Dairy and gluten in disease activity in multiple sclerosis. Mult Scler J Exp Transl Clin. 2023;9(4):20552173231218107.

[37] Zevallos VF, Yogev N, Hauptmann J, Nikolaev A, Pickert G, Heib V, et al. Dietary wheat amylase trypsin inhibitors exacerbate CNS inflammation in experimental multiple sclerosis. Gut. 2023;73(1):92–104.
[38] Mannino A, Daly A, Dunlop E, Probst Y, Ponsonby AL, van der Mei IAF, et al. Higher consumption of ultra-processed foods and increased likelihood of central nervous system demyelination in a case-control study of Australian adults. Eur J Clin Nutr. 2023;77(5):611–4.
[39] Guglielmetti M, Grosso G, Ferraris C, Bergamaschi R, Tavazzi E, La Malfa A, et al. Ultra-processed foods consumption is associated with multiple sclerosis severity. Front Neurol. 2023;14:1086720.
[40] Kleinewietfeld M, Manzel A, Titze J, Kvakan H, Yosef N, Linker RA, et al. Sodium chloride drives autoimmune disease by the induction of pathogenic TH17 cells. Nature. 2013;496(7446):518–22.
[41] Miclea A, Bagnoud M, Chan A, Hoepner R. A brief review of the effects of vitamin D on multiple sclerosis. Front Immunol. 2020;11:781.
[42] Ghaseminejad-Raeini A, Ghaderi A, Sharafi A, Nematollahi-Sani B, Moossavi M, Derakhshani A, et al. Immunomodulatory actions of vitamin D in various immune-related disorders: A comprehensive review. Front Immunol. 2023;14:950465.
[43] Balasooriya NN, Elliott TM, Neale RE, Vasquez P, Comans T, Gordon LG. The association between vitamin D deficiency and multiple sclerosis: An updated systematic review and meta-analysis. Mult Scler Relat Disord. 2024;90:105804.
[44] Kousparou C, Fyrilla M, Stephanou A, Patrikios I. DHA/EPA (Omega-3) and LA/GLA (Omega-6) as bioactive molecules in neurodegenerative diseases. Int J Mol Sci. 2023;24(13).
[45] AlAmmar WA, Albeesh FH, Ibrahim LM, Algindan YY, Yamani LZ, Khattab RY. Effect of omega-3 fatty acids and fish oil supplementation on multiple sclerosis: A systematic review. Nutr Neurosci. 2021;24(7):569–79. doi: 10.1080/1028415X.2019.1659560.
[46] Beckett JM, Bird ML, Pittaway JK, Ahuja KD. Diet and multiple sclerosis: Scoping review of web-based recommendations. Interact J Med Res. 2019;8(1):e10050.
[47] Birnbaum G, Stulc J. High dose biotin as treatment for progressive multiple sclerosis. Mult Scler Relat Disord. 2017;18:141–3. doi: 10.1016/j.msard.2017.09.030.
[48] Torkildsen O, Wergeland S, Bakke S, Beiske AG, Bjerve KS, Hovdal H, et al. ω-3 fatty acid treatment in multiple sclerosis (OFAMS Study): A randomized, double-blind, placebo-controlled trial. Arch Neurol. 2012;69(8):1044–51.
[49] Mauriz E, Laliena A, Vallejo D, Tuñón MJ, Rodríguez-López JM, Rodríguez-Pérez R, et al. Effects of a low-fat diet with antioxidant supplementation on biochemical markers of multiple sclerosis long-term care residents. Nutr Hosp. 2013;28(6):2229–35.
[50] Moravejolahkami AR, Chitsaz A, Hassanzadeh A, Paknahad Z. Anti-inflammatory-antioxidant modifications and synbiotics improved health-related conditions in patients with progressive forms of multiple sclerosis: A single-center, randomized clinical trial. Complement Ther Clin Pract. 2023a;53:101794.
[51] Moravejolahkami AR, Chitsaz A, Hassanzadeh A, Paknahad Z. Effects of anti-inflammatory-antioxidant-rich diet and co-supplemented synbiotics intervention in patients with progressive forms of multiple sclerosis: A single-center, single-blind randomized clinical trial. Nutr Neurosci. 2023b;26(11):1078–89.
[52] Guan JZ, Guan WP, Maeda T. Vitamin E administration erases an enhanced oxidation in multiple sclerosis. Can J Physiol Pharmacol. 2018;96(11):1181–3.
[53] Rudrapal M, Khairnar SJ, Khan J, Dukhyil AB, Ansari MA, Alomary MN, et al. Dietary polyphenols and their role in oxidative stress-induced human diseases: Insights into protective effects, antioxidant potentials and mechanism(s) of action. Front Pharmacol. 2022;13:806470.
[54] Dolati S, Ahmadi M, Aghebti-Maleki L, Nikmaram A, Marofi F, Rikhtegar R, et al. Nanocurcumin is a potential novel therapy for multiple sclerosis by influencing inflammatory mediators. Pharmacol Rep. 2018;70(6):1158–67.
[55] Dolati S, Babaloo Z, Ayromlou H, Ahmadi M, Rikhtegar R, Rostamzadeh D, et al. Nanocurcumin improves regulatory T-cell frequency and function in patients with multiple sclerosis. J Neuroimmunol. 2019;327:15–21.
[56] Petracca M, Quarantelli M, Moccia M, Vacca G, Satelliti B, D'Ambrosio G, et al. Prospective study to evaluate efficacy, safety and tolerability of dietary supplement of curcumin (BCM95) in subjects with active relapsing multiple sclerosis treated with subcutaneous interferon beta 1a 44 mcg TIW (CONTAIN): A randomized, controlled trial. Mult Scler Relat Disord. 2021;56:103274.
[57] Means JC, Lopez AA, Koulen P. Resveratrol protects optic nerve head astrocytes from oxidative stress-induced cell death by preventing caspase-3 activation, tau dephosphorylation at ser(422) and formation of misfolded protein aggregates. Cell Mol Neurobiol. 2020;40(6):911–26.
[58] Villar-Delfino PH, Santos RP, Christo PP, Nogueira-Machado JA, Volpe CMO. Antioxidant effects of resveratrol in granulocytes from multiple sclerosis patients. Explor Neurosci. 2024;3:362–74. doi: 10.37349/en.2024.00055.
[59] Koushki M, Dashatan NA, Meshkani R. Effect of resveratrol supplementation on inflammatory markers: A systematic review and meta-analysis of randomized controlled trials. Clin Ther. 2018;40(7):1180–92.e5.
[60] Hossen I, Kaiqi Z, Hua W, Junsong X, Mingquan H, Yanping C. Role of polyphenols in the management of neurodegenerative diseases: Alzheimer's and Parkinson's. Nutrients. 2022;14(3):620. doi: 10.3390/nu14030620.
[61] Rust R, Chien C, Scheel M, Brandt AU, Dörr J, Wuerfel J, et al. Epigallocatechin gallate in progressive MS: A randomized, placebo-controlled trial. Neurol Neuroimmunol Neuroinflamm. 2021;8(3).
[62] Sedel F, Papeix C, Bellanger A, Touitou V, Lebrun-Frenay C, Galanaud D, et al. High doses of biotin in chronic progressive multiple sclerosis: A pilot study. Mult Scler Relat Disord. 2015;4(2):159–69.
[63] Tourbah A, Lebrun-Frenay C, Edan G, Clanet M, Papeix C, Vukusic S, et al. MD1003 (high-dose biotin) for the treatment of progressive multiple sclerosis: A randomised, double-blind, placebo-controlled study. Mult Scler. 2016;22(13):1719–31.
[64] Couloume L, Barbin L, Leray E, Wiertlewski S, Le Page E, Kerbrat A, et al. High-dose biotin in progressive multiple sclerosis: A prospective study of 178 patients in routine clinical practice. Mult Scler. 2020;26(14):1898–906.
[65] Miyake S, Kim S, Suda W, Oshima K, Nakamura M, Matsuoka T, et al. Dysbiosis in the gut microbiota of patients with multiple sclerosis, with a striking depletion of species belonging to clostridia XIVa and IV clusters. PLoS One. 2015;10(9):e0137429.
[66] Stoiloudis P, Kesidou E, Bakirtzis C, Sintila SA, Konstantinidou N, Boziki M, et al. The role of diet and interventions on multiple sclerosis: A review. Nutrients. 2022;14(6).

[67] Berer K, Gerdes LA, Cekanaviciute E, Jia X, Xiao L, Xia Z, et al. Gut microbiota from multiple sclerosis patients enables spontaneous autoimmune encephalomyelitis in mice. Proc Natl Acad Sci U S A. 2017;114(40):10719–24.
[68] Tremlett H, Fadrosh DW, Faruqi AA, Zhu F, Hart J, Roalstad S, et al. US network of pediatric MS centers. Gut microbiota in early pediatric multiple sclerosis: A case-control study. Eur J Neurol. 2016;23(8):1308–21. doi: 10.1111/ene.13026.
[69] Moles L, Otaegui D. The impact of diet on microbiota evolution and human health. Is diet an adequate tool for microbiota modulation? Nutrients. 2020;12(6).
[70] Lombardi VC, De Meirleir KL, Subramanian K, Nourani SM, Dagda RK, Delaney SL, et al. Nutritional modulation of the intestinal microbiota; future opportunities for the prevention and treatment of neuroimmune and neuroinflammatory disease. J Nutr Biochem. 2018;61:1–16.
[71] Kouchaki E, Tamtaji OR, Salami M, Bahmani F, Daneshvar Kakhaki R, Akbari E, et al. Clinical and metabolic response to probiotic supplementation in patients with multiple sclerosis: A randomized, double-blind, placebo-controlled trial. Clin Nutr. 2017;36(5):1245–9.
